# AN ANCIENT DNA PACIFIC JOURNEY: A CASE STUDY OF COLLABORATION BETWEEN ARCHAEOLOGISTS AND GENETICISTS

**DOI:** 10.1080/00438243.2019.1733069

**Published:** 2020-03-17

**Authors:** Matthew Spriggs, David Reich

**Affiliations:** 1 Vanuatu National Museum, Vanuatu Cultural Centre, P.O. Box 184, Port Vila, Vanuatu; 2 Department of Genetics, Harvard Medical School, Boston, MA 02115, USA; 3 Howard Hughes Medical Institute, Boston, MA 02115, USA; 4 Medical and Population Genetics Program, Broad Institute of MIT and Harvard, Cambridge, MA 02142, USA; 5 Department of Human Evolutionary Biology, Harvard University, Cambridge, MA 02138, USA

**Keywords:** Ancient DNA, palaeogenomics, collaboration between archaeologists and geneticists, interdisciplinary perspectives

## Abstract

We present a case-study of a collaboration between archaeologists and geneticists that has helped settle a long-standing controversy and opened up new research questions for the Pacific region. The work provided insights into the history of human settlement and cultural changes in Vanuatu in the western Pacific, which in turn shed light on the origins of the cultural and linguistic diversity that characterizes the archipelago. Close interdisciplinary collaborations like this maximize the potential of ancient DNA to contribute to our understanding of the past and advance the scholarship of practitioners in both disciplines.

There has been much recent discussion of the evolving and increasingly interdependent relationships between geneticists and archaeologists as the whole genome aDNA revolution gathers pace (for recent reviews see Piscitelli (ed.) 2019 and Vereemah 2018). The interaction, as can be seen from the papers in this volume, has raised the quality of work in both disciplines. Here we take a case-study approach to this topic, documenting our ancient DNA collaborative journey in the western Pacific. We hope that a detailed example of work in progress will provide useful perspectives for those contemplating interdisciplinary cooperation across two traditions with different histories and styles and expectations.

In the Western Pacific today, Melanesians and Polynesians have dual ancestry from Papuans and populations that ultimately have an East Asian origin. In the genetic sense ‘Papuan’ is a shorthand for the shared ancestry that links peoples of Northern Sahul, the Bismarck Archipelago and the main Solomon Islands chain (the region of ‘Near Oceania’) who arrived there about 50,000BP, and who are classically represented today by New Guinea Highlands populations. They show deep genetic affinities with Indigenous Australian groups of southern Sahul, a region settled at about the same time as part of an early ‘Out of Africa’ movement of modern humans (see Kirch 2017: Chapter 3 for a recent summary of the Pleistocene archaeology of the region). The proportion of ancestors who are East Asian-related is in general much greater in Polynesian populations than it is in in Near Oceania and in Vanuatu, New Caledonia and Fiji in Remote Oceania. It has been argued that the East Asian-related ancestry is related to the spread of the Lapita culture (Kirch 2017: Chapter 4), as an eastwards extension of Southeast Asian Neolithic cultures, ultimately from Taiwan and southern China (Figure 1). The first evidence of the Lapita culture is in the Bismarck Archipelago sometime around 3350-3100BP, and it then apparently leapfrogged the main Solomon Islands in its earlier phases of eastward and southern expansion (Sheppard 2011). Lapita thus represents the first peopling of Remote Oceania from about 3000BP in the Reefs-Santa Cruz Group, Vanuatu, New Caledonia and Fiji, and a bit later (2850BP) in Tonga and then Samoa (2750BP) (see references in Petchey et al. 2015).

There are good archaeological reasons to believe that the earliest phase of Remote Oceanic human occupation has been identified, in the form of a rolling wave of faunal extinctions and local extirpations across the region within a few hundred years of human occupation (Steadman 2006). In Vanuatu, for example (Figure 2), the earliest archaeological sites include bones of giant tortoises (Hawkins et al. 2016; genus subject to revision), land crocodiles (Mead et al. 2002) and extinct bird species such as a very large megapode, *Mwalau walterlini* (Worthy et al. 2015), which are all no longer present. Shellfish in the earliest sites are often much larger than specimens found today, a point often noted by Indigenous members of the excavation teams (cf. Piroutet 1909:609 for the eponymous site of Lapita in New Caledonia).

The Teouma cemetery is located just outside Vanuatu’s capital Port Vila on Efate Island. It was excavated between 2004 and 2010 in six field seasons as a joint project of The Australian National University (ANU) and the Vanuatu Cultural Centre/Kaljoral Senta (VKS), working with Ni-Vanuatu villagers and Australian and French field school crews (for general accounts see Bedford et al. 2006, 2009, 2010; Spriggs and Bedford 2013; Valentin et al. 2010).^1^ A significant public outreach program included a 2009–10 AUSAID-funded schools program exposing both Primary and High School students from local schools to archaeological methods (Shing 2013). The excavation had the full support of the traditional Eratap village landowners and chiefs, and of the leaseholder Robert Monvoisin.

Teouma is the largest and so-far earliest Lapita culture cemetery known in the Pacific, with 68 burial features and just over 100 individuals, displaying a variety of body treatments including removal of all skulls and a variety of other bones of adult individuals, secondary burials, including inside Lapita pots, a cremation, and a variety of body positions including both flexed and extended burials. A small number of skulls and mandibles were placed on the chests or between the legs of other individuals, and in one case three mandibles were placed under a pile of long bones. Some burials were accompanied by Lapita pots, and very occasionally had *Conus* shell anklets (Langley et al. 2019). Offerings of joints of pork and even in one case a tortoise carapace were placed with burials and the missing skulls were sometimes replaced by *Conus* shell rings and/or slabs of rock (Valentin et al. 2010).

From early on in the project, attempts were made to extract DNA but the technologies available at the time were not up to the task. Three university laboratories in three countries failed to produce ancient DNA data that passed standard criteria for authenticity (that is, the DNA sequences produced were indistinguishable from what would be expected from modern contamination).^2^ The problems of extracting aDNA in tropical conditions appeared overwhelming and this avenue of research seemed closed. Alternative methods of examining the origins of the Teouma skeletons provided the first meaningful progress in using bioarchaeological approaches to understanding the Teouma site (Bentley et al. 2007). Strontium analysis was suggestive of some of the people buried at the site being migrants from elsewhere but was not specific as to place of origin, not least because of the then-total lack of baseline studies in the Pacific region and reliance on geological values. The Teouma project began to rectify this situation and there are now baseline values from throughout Vanuatu and from several adjoining island groups: Fiji, New Caledonia and the Solomon Islands, but as yet not from anywhere in the proximate Lapita ‘homeland’ of the Bismarck Archipelago just to the east of New Guinea, where only geological values are available.^3^

In this paper, we present a synthesis of what the three key ancient DNA studies of Remote Oceania to date have shown. We also recount some of the discussions that arose during the work carried out for these studies, as a way of highlighting the challenges and promise of collaborations between archaeologists and geneticists. We conclude by putting our ancient DNA research program in Vanuatu into a broader context by comparing it to several other collaborations between archaeologists and geneticists in which one of us has been involved (D.R.). The Vanuatu experience is an instance of an increasingly rich set of collaborations in which archaeologists and geneticists are equal partners and that study-by-study are helping to move both fields in positive directions.

## Skoglund et al. 2016: Discovery that First Remote Oceanians Had Little Papuan Ancestry

In 2015 the project directors Matthew Spriggs and Stuart Bedford were approached by Ron Pinhasi, who is a close collaborator of one of us (D.R.), and whose team had recently identified the petrous bone of the skull as a potential source for relatively well-preserved aDNA even in tropical areas (Gamba et al. 2014; Pinhasi et al. 2015). Our collaborative team then proceeded to successful extraction, analysis and publication of data from three individuals (Skoglund et al. 2016). This was the first whole-genome ancient DNA data published from the tropics and was also notable in a field that until then had been focused on Europe. The paper also added data from a fourth individual, represented by a petrous bone from the Talasiu site on Tongatapu that was part of a separate project on Tongan prehistory led by Johannes Krause, Cosimo Posth and colleagues at the Max Planck Institute for the Science of Human History, Jena, Germany, and that dated either to the latest Lapita phase occupation or immediately post-Lapita at c. 2650BP. We added this extremely significant sample to the paper – extending the range of geographic coverage of early aDNA samples by ~2000 km into geographical Polynesia – after the first round of review, once we found out from the Jena team that they had independently processed it and after our two groups agreed that combining the samples could make for a more compelling final paper. Such combining of data produced by independent research groups and adding new data into resubmitted papers is common in the molecular biology community, the academic culture from which the field of ancient DNA emerged. ^4^

There have long been two major opposed theories as to why there are significant phenotypic differences between Melanesians of both Near and Remote Oceania and Remote Oceanic Polynesians. One theory was that people of ultimately East Asian ancestry moved into the Bismarck Archipelago and encountered and mixed with Papuan populations there to various degrees. In this scenario, groups with more admixture reached the Reefs-Santa Cruz, Vanuatu and New Caledonia, and those with less admixture went on to Fiji, Tonga and Samoa to form, eventually, a distinctly Polynesian population (Kayser 2010; Wollstein et al. 2010). Fiji has always been seen as a border area between the two groups, although today it is linguistically distinct from Polynesia (Clark 2003a, 2003b). Subsequent admixture within the Solomon Islands, perhaps in Late Lapita times when a secondary spread of Lapita there can be detected, extended the admixed pattern of the earlier migrating groups (Walter and Sheppard 2017). This theory became substantially more popular on the basis of studies of DNA of present-day people, initially mitochondrial DNA (mtDNA), then Y Chromosome and whole genome analysis. It was shown that all present-day Indigenous Remote Oceanians had substantial Papuan-related ancestry, which was taken as evidence that the first people of the Remote Pacific were already heavily mixed, reflecting an extensive and intensive period of social interaction with Papuan-related populations they encountered prior to ~3000BP (Kayser 2010; Wollstein et al. 2010; Matisoo-Smith 2015).

The alternative (and older) theory was that people of ultimate East Asian ancestry moved through the Bismarck Archipelago without much admixture, settling Remote Oceania as far as Tonga and Samoa, and only later did a secondary movement of people with a high proportion of Papuan ancestry spread throughout this region. Papuan-related ancestry and phenotypes attenuated further east, reflecting increasing dilution through admixture with the previously established populations of mostly East Asian ancestry. This serial dilution would explain the findings based on present-day DNA that Polynesians harbour a low proportion of Papuan-related genetic ancestry (around 25% based on the most up-to-date estimates, Skoglund et al. 2016). On the other hand, Remote Oceanic groups as far east as Fiji retained generally more than 50% of that ancestry, with the highest proportions in Vanuatu of around 90%. Within this theory, the question of whether this was a second wave of settlement or a slower process of continuing gene flow over a long period was also debated (Friedlaender et al. 2008; Spriggs 1997:158–9).

The second theory was favoured by the ancient DNA results from Vanuatu and Tonga, which revealed that people with little or no Papuan ancestry were present among the initial inhabitants of both archipelagos. The extremely low proportion of Papuan ancestry (the estimate for Teouma in Skoglund et al. 2016 was 0–11%, which was reduced and tightened to 0.1–4.7% with the higher resolution data and analysis presented in a later paper by Lipson et al. 2018 – see below) is far outside the range in all present-day Remote Oceanians who have ~25–90% Papuan ancestry, implying that the thorough intermixing of Papuan ancestry into all the peoples of this vast region must have occurred later.

The focus of Skoglund et al. (2016) was on presenting a clear genetic result, which would inspire further questions, such as the reasons for the subsequent spread of Papuan ancestry across the region over time (to a massive degree in Vanuatu and to a lesser degree in Tonga) – questions to be addressed in future work. Archaeologist co-authors contributed to the study by framing the questions, sharing the samples, explaining the significance of the results, and critically reviewing the language of the manuscript to ensure that appropriate terminologies were used to describe the people whose remains were being studied, especially given past elision of categories derived from other disciplines to describe genetic findings in the Pacific. After extensive debate, we coined the term ‘First Remote Oceanians’ to describe the East Asian-related ancestry that we detected as spreading early to Remote Oceania. We chose to use this term, rather than alternative possibilities like ‘Lapita’ or ‘Austronesian’ or ‘East Asian’, because it implied nothing about cultural or linguistic affiliation or geographic origin—all important issues, but ones that we did not have the data to settle at the time we wrote the paper (for discussion of this general problem of terminology in ancient DNA studies, see Eisenmann et al. 2018).

While the Skoglund et al. (2016) ancient DNA findings disproved the prevailing theory in the genetic literature (Kayser 2010; Wollstein et al. 2010; Matisoo-Smith 2015), to many of the archaeologist co-authors the ancient DNA-based support for the second theory was unsurprising. For example, a paper published earlier in the same year by Valentin and colleagues (2016) showed changes in skeletal morphology comparing the Lapita skeletons in Teouma to later skeletons in Vanuatu and across the Pacific, and argued on this basis and in concert with archaeological evidence that there was large-scale gene flow into Vanuatu in the Late Lapita or immediately post-Lapita period. As that paper pointed out in its final paragraph, archaeological evidence relating to how and when people with this ancestry first appeared and from whence they came would have to await more data from later periods (see also Valentin et al. 2014).

Pacific archaeologists are used to vigorous debate with their peers. The first draft of the paper benefited from some particularly critical reviews, the most substantial issue of which concerned whether the ancestry of the three individuals from Teouma was common in Lapita or instead was a specific feature of the individuals analysed from the site. These questions were substantially answered by the inclusion in the final version of the paper of the Tongan sample processed in Germany from a completely separate project, which greatly increased the geographic scope and provided a picture entirely similar to that at Teouma. It was already clear from Skoglund et al. (2016) that people of almost entirely East Asian-related ancestry were widespread and common across Remote Oceania in the Lapita and immediately post-Lapita periods, a sharp contrast to today when there are no Indigenous populations with this ancestry profile except perhaps in Micronesia which has a more complex archaeological history involving settlement from multiple origin points (Kirch 2017: Chapter 6). Something dramatic must have occurred between the Lapita period and the present to further transform the genetic landscape of Remote Oceania.

## Lipson et al. 2018: Major Papuan Gene Flow into Vanuatu at the End of the Lapita Period

One aspect of the Skoglund et al. (2016) study that left the participating archaeologists perplexed was the genetic estimates of when admixture had occurred between First Remote Oceanians and Papuans. In Skoglund et al. (2016), the dates for admixture were estimated to have occurred on average 2300–1500 BP based on the average sizes of stretches of First Remote Oceanian ancestry interspersed with Papuan ancestry in the genomes of living people (after initial admixture, stretches of DNA from the two ancestry sources are diced up at a regular rate every generation, so their average size in present-day people carries information about how long ago mixture occurred). The genetic estimates for the admixture dates were consistent with ones obtained by a similar method in an earlier study which included present-day Remote Oceanian DNA (Lipson et al. 2014), but were significantly younger than the Lapita or even possible pre-Lapita-era dates produced in an earlier study using a different statistical method (Xu et al. 2012).

However, the average dates of 2300–1500 BP were if anything too recent for the archaeologists, because they were after the end of the Lapita period, which seemed too late given archaeological knowledge of Vanuatu’s material culture history and extra-archipelago connections. Archaeologically, there is no evidence for any continued contacts across the Near to Remote Oceania boundary post-Lapita until about the last 1000 years – that is, the archaeology suggests a hiatus in extra-archipelagic contacts from the end of the Lapita period in the mid-third millennium BP until the beginning of the last millennium BP – and thus it would be surprising for there to be major gene flow into the Vanuatu archipelago in this extended period of one to two millennia. For the archaeologists, the most likely period of secondary movement of people into Vanuatu was during or at the end of the Lapita phase itself, which finished in central and southern Vanuatu by about 2800BP and on Malakula in the north some time later at around 2400BP, albeit for the last 400 years without direct evidence of extra-archipelagic contacts (cf. Spriggs et al. 2019:53–4).

To unpack this archaeological evidence in more detail – and to explain why the end of the Lapita period would have been a natural time for the large-scale arrival of Papuan ancestry in Vanuatu but not the millennium or two afterward – at the end of the Lapita period there was not only an end to long-distance artefact exchange, but also what has been called a ‘egalitarian turn’ (Earle and Spriggs 2015: 523) whereby archaeological evidence for social hierarchies becomes nearly invisible. Recent studies have explicitly hypothesized that this cultural change might reflect new people with different social structures spreading through Remote Oceania and perhaps even contributing to the end of the Lapita culture (Earle and Spriggs 2015; Valentin et al. 2014). If the genetic dates of admixture were after this first period, they would only make sense if mixture between people of Papuan and First Remote Oceanian ancestry substantially post-dated their time of first contact. In other words, to reconcile the evidence from archaeology and genetics, Remote Oceania would have had to be a patchwork of peoples with higher and lower proportions of Papuan ancestry due to social or geographic segregation for hundreds of years after the secondary Papuan migrations occurred, and before the groups homogenized genetically. If this occurred, then the dates of mixture would significantly post-date the time of migration, as observed.

To address this discrepancy and provide additional information about the timing of the secondary migration that brought Papuan ancestry, one of the Teouma team’s bioarchaeologists, Fréderique Valentin, proposed an aDNA time transect in central Vanuatu, particularly Efate Island, analysing skeletal samples from a range of dated sites. The goal was to examine the proportion of admixture over time and measure how and when it changed. A further goal of this study was to address the limitation that none of the 778 present-day DNA samples analysed for the Skoglund et al. (2016) paper was from Vanuatu itself; instead, the comparison areas in the Pacific were New Guinea, the Bismarck Archipelago, Solomon Islands including Santa Cruz, Polynesian Outliers in the Solomon Islands, and Tonga. It was important to obtain present-day comparative samples from across Vanuatu, and for this purpose we collaborated with colleagues at the University of Oxford whose laboratory beginning in the 1970s had collected DNA samples from throughout the Pacific to address a range of research questions about human variation.

In 2014, the University of Oxford institutional review board carried out an independent ethics review to address the question of whether the permissions associated with the Pacific samples in their collections were consistent with broad studies of human population history given the informed consent standards in place at the time the samples were collected, and it was determined that this was the case. To add an extra layer of confidence about whether it was appropriate to study these samples specifically in the context of our ancient DNA study, we carried out consultations with national institutions in Vanuatu itself, both with the Vanuatu Cultural Centre (which incorporates the National Museum) and at the ministerial level within the Government of Vanuatu. Explicit approval for the use of the anonymised archived samples for the purpose of studying population history was given in a letter from the Vanuatu Cultural Centre in May 2017. With these permissions in place, Lipson et al. (2018) reported whole-genome data from 185 present-day Ni-Vanuatu individuals from 18 islands and a total of 34 populations, providing a new resource for understanding the population structure of the archipelago today and its relationship to that of ancient people.

The Lipson et al. (2018) study did not manage to obtain working ancient DNA data from all time periods from 3000BP to the European contact era; either samples of the required dates produced no ancient DNA or there were simply no samples available from key time periods. Thus, the study included early samples associated with the Lapita culture from Teouma, around 2900BP (increasing the quality and extent of the data from the previous study). The next oldest sample that produced results was from the site of Taplins on Efate at 2300BP, meaning there were no successful samples from the crucial period of Papuan arrival that the study had set out to investigate. There was another approximately 1000-year gap before the next samples at around 1300BP from the site of Burumba on Epi Island to the north of Efate, then another approximately 800-year gap to a sample at the site of Mangaliliu on Efate of 500BP and a further six samples from Efate and Epi that covered the last 500 years (Lipson et al. 2018).

These gaps were significant but did not mean that the study could not produce meaningful constraints on what occurred in the intervening periods. This was because the oldest ancient DNA sample with a high proportion of Papuan ancestry provided a *terminus ante quem* minimum date for the arrival of Papuan ancestry in Vanuatu; because admixture estimates could now be calculated based on samples at intervals along the 3000-year timeline; and because comparisons could be made with the 185 present-day Vanuatu individuals. The analysis showed that Papuan ancestry was definitely in Vanuatu by ~2300BP (the date of the Taplins individual, who was almost entirely Papuan in ancestry), and that the admixture began minimally hundreds of years before the date of this sample. Thus, the data were consistent with Papuan admixture occurring in the Late Lapita or immediately Post Lapita period (in line with the archaeological constraints on the time of plausible movement from Near Oceania). The analysis furthermore showed that the admixture must have continued after the time that gene flow began as groups with different proportions of Papuan ancestry mixed within an archipelago that had become highly substructured from the perspective of material culture (and thus plausibly included barriers to social mixing as well) – a pattern of substructure that persists today in the extraordinary number of distinct languages in Vanuatu per head of population. There was also a hint that some of the younger dates that had been produced might relate to a more recent third movement of people, which introduced Polynesian languages and genes to Vanuatu and led to the so-called Polynesian Outlier communities dating to within the last thousand years (Flexner et al. 2019; Spriggs 1997: Chapter 7).

## Posth et al. 2018a: Independent Insights About Papuan Gene Flow in the Late Lapita Period

The results became available to the core members of the team in mid-2017, but final drafting of the paper was delayed by the move of Pontus Skoglund from Harvard to London to set up a new laboratory at the Francis Crick Institute (he had been leading our team’s genetic analysis up until that time). Meanwhile a team based at the Max Planck Institute for the Science of Human History in Jena Germany, also working with the Vanuatu Cultural Centre but on separate research projects, had developed another Vanuatu ancient DNA study based on archaeologicfal samples from Malakula in the north and Tanna and Futuna in southern Vanuatu, as well as additional aDNA samples from Tonga, French Polynesia and an ancient sample from Malaita in the Solomon Islands (published as Posth et al. 2018a). Some of the same archaeologists were authors on both papers.

While some early discussion was held as to the possibility of combining both projects into a single paper, authorship issues when combining results from two large teams of researchers proved intractable in this particular case. There was also a desire to keep the two studies separate among some of the archaeologists involved (including M.S.), who thought it appropriate to have two separate genetics teams independently examining closely related sets of questions, with the goal of increasing confidence in any consistent findings and identifying areas of inconsistency. A similar approach of dividing samples across groups for other bioarchaeological analysis—for example isotopic analysis and radiocarbon dating—had been pursued by the Vanuatu archaeologists since the start of the Teouma project in 2004. It has proved useful in evaluating novel techniques and applications, as well as different pretreatment methodologies.

There were also somewhat different approaches to presenting the evidence between the two teams, even while both teams had as their ultimate intellectual goal a synthesis of the archaeological and genetic and linguistic data. The philosophy of the Lipson et al. (2018) team was to write a paper that would neutrally report the genetic findings as a documentation of the genetic evidence for all scholars in archaeology and linguistics who might have alternative theories about the implications. The Posth et al. (2018a) team followed a different approach, explicitly focusing on synthesizing lines of evidence from genetics and other fields, a philosophy that was evident in their title ‘Language continuity despite population replacement in Remote Oceania’ as well as the additional archaeological and ethnographic analyses included within the core interpretative scheme they used.

Among the archaeologists there were contrasting views on which of these two approaches was better. Papers that are true syntheses of genetic findings with sophisticated argumentation based on new data from linguistics and archaeology are important. Posth et al. (2018a) was an example of this. At the same time, the potential pitfalls associated with this approach in the context of the primary reporting of the genetic data are evident in some of the reactions to Posth et al. (2018a). The genetic findings were crystal-clear and were almost entirely consistent with those in Lipson et al. (2018). At the same time, the Posth et al. (2018a) findings provided additional richness by showing with multiple samples from the immediate post-Lapita period that there was an extended period of mixture between people of First Remote Oceanic and Papuan ancestry at least in northern Vanuatu.

A comparison of the results of the two papers added further insights by showing that the dynamics of population change after the Lapita period were different in Malakula in the north of the archipelago (sampled in Posth et al. (2018a)) compared to both Efate/Epi in the centre of the archipelago (sampled in Lipson et al. (2018)) and Tanna and Futuna in the south of the archipelago (sampled in Posth et al. (2018a)). Specifically, high proportions of First Remote Oceanian ancestry lasted hundreds of years later in Malakula than in the other islands, with Malakula also notably being an island where characteristically Lapita material culture also lasted for many additional centuries. However, the linguistic ideas that were drawn upon in Posth et al. (2018a) have been contested by some professional linguists (Naess and Pawley in Bedford et al. 2018) and some geneticists knowledgeable about Pacific linguistic arguments (Cox in Bedford et al. 2018), who argued that it is possible that the Austronesian languages spoken on Vanuatu today could have come to the archipelago along with the late Lapita Papuan migrants, and thus there may not have been linguistic continuity. This meant that the reception of the paper’s genetic findings was complicated by advancing a synthesis of genetics with other lines of evidence that some felt went beyond what could be supported (see Posth et al. 2019: 59–60 for a response to the criticisms by Naess, Pawley and Cox).

An example of a choice that Posth et al. (2018a) made that differed from the choice made in Lipson et al. (2018) and Skoglund et al. (2016) and that could potentially be the subject of contention is the use of the language family term ‘Austronesian’ to describe the East Asian-related migrant group, a group that was referred to as ‘First Remote Oceanians’ in Skoglund et al. (2016) and later in Lipson et al. (2018). As discussed above, the coining of the term ‘First Remote Oceanians’ was the product of discussions between the geneticist and archaeologist co-authors that occurred in the context of writing Skoglund et al. (2016), and came about because of a decision that it is better to decouple linguistic classifications from genetic ones even if they may sometimes coincide, because people with any ancestry background can and do learn to speak new languages. Similarly, the use of hybrid terms such as ‘Lapita-Austronesian’ (later elided as ‘Lapita ancestry’ in discussion of present-day Tongans in Posth et al. (2018a:734)) is potentially confusing – what if some bearers of the Lapita culture were found to be primarily Papuan-related?

We share the general concern of Johanssen et al. (2017:1119) that: ‘We cannot assume that individuals who shared material culture traits were part of the same biological population: One can have similar traits without relatedness (owing to convergence or exchange) and relatedness without similarity in traits (owing to divergence) … Equally, language groups cannot necessarily be conflated with biological populations, material assemblages, or even social units’. Posth et al. (2018a) fully agree that there is no one-to-one mapping between language and genetics, as is highlighted by the very title of their paper. To avoid the possibility of confusion, and in line with a recent recommendation for naming of groups in genetic studies (Eisenmann et al. 2018), we advocate the use of specifically genetic terms for genetic entities (First Remote Oceanian, Papuan),^5^ cultural terms for cultural entities (Lapita) and linguistic terms for linguistic entities (Austronesian/Non-Austronesian).

## How Genome-Wide Ancient DNA from Vanuatu Advanced the Discussion in Archaeology

The main criticism made of Lipson et al. (2018) was the opposite of the critique of Posth et al. (2018a): Kirch suggested that its authors minimized discussion of the archaeological evidence ‘because they regard archaeology as irrelevant to the story they are telling’ (Kirch, in Bedford et al. 2018:210). However, this was not the case; the writing of the paper instead followed a principled approach that was supported by the archaeologist co-authors. Far from ignoring the archaeological evidence, we took seriously the position advocated previously by Kirch and Green (2001) that ‘triangulation’ among different disciplines involved in documenting the Pacific deep past required initial independence of the lines of evidence being compared. Lipson et al. (2018) presented one such line of evidence. Following a useful set of commentaries on both the Lipson et al. (2018) and Posth et al. (2018a) papers in a forum in the journal *Archaeology in Oceania* (Bedford et al. 2018), we wrote a response that sought to explore some of the points of triangulation between the genetic evidence and other disciplines (Spriggs et al. 2019). The archaeologists took the lead on this latter paper as most of the critical commentary related to points of archaeology rather than of genetics. The Jena-based team also wrote a response, which focused heavily on the archaeological and linguistic issues in light of the genetic findings (Posth et al. 2019).

One incorrect statement in the forum was that the three aDNA papers ‘required a complete reassessment of the process of Lapita expansion, particularly regarding the degree of interaction in Near Oceania during the 300-plus years of occupation prior to populations moving out into Remote Oceania’ (Matisoo-Smith, in Bedford et al. 2018:211). However, as discussed above, prior to the ancient DNA papers there were in fact two major alternative models for the human process that spread the Lapita culture, neither of which could fairly be described as the ‘consensus’ view. What the three papers did was to reject one model, and then to add additional details that could only be obtained by taking advantage of the unique information provided by genome-wide ancient DNA data.

The vigorous and thoughtful commentary in the Bedford et al. (2018) forum gives the lie to the idea that archaeologists and linguists were either excluded from a discussion driven by genetics or that their evidence was disregarded by geneticists. But it does reveal the contrasting styles of argumentation in genetics compared to archaeology. Genetic models that seek to carry out formal hypothesis testing can seem overly simplistic to archaeologists dealing with complex processes influencing cultural change and indeed population history as well. But more complex archaeological models often come at the price of being framed in ways that are untestable, and therefore more subjective. We believe that both approaches have an important role. Specifically, the fact that genetic data can highlight connections between peoples not previously known to be in strong contact—and can also identify cases of limited gene flow—can provide valuable constraints on archaeological models. Far from promoting over-simplified models, genetic studies offer the opportunity to collect data that can form the basis for more complex ones. Thus, based on ancient DNA data, there is now irrefutable evidence for at least three independent prehistoric movements of people that have had major demographic impacts on Vanuatu: (1) initial peopling by a First Remote Oceanian population of almost entirely East Asian ancestry, (2) the spread of Papuan ancestry into Vanuatu in the Late Lapita or immediately Post-Lapita period and admixture into the local population with dynamics that differed across islands, and (3) gene flow from Polynesia that affected different parts of Vanuatu in different ways, a topic that is the focus of our most recent collaborative research (discussed in the next section). Knowing about these three population formation events provides a basis for asking more sophisticated questions about the processes that contributed to each of them.

## Vanuatu as an Exemplar of Close Collaboration Between Archaeologists and Geneticists

As will be clear to readers of this paper, our collaboration is an ongoing one, representing a deep and long-term commitment on the part of the archaeologists and geneticists involved. Rather than stopping with the discovery that the First Remote Oceanians fell outside the genetic variation of present-day Remote Oceanians (Skoglund et al. 2016)—a finding that was disruptive to the literature and accordingly was published in a higher profile journal *(Nature*) than any of our later work on this topic—we and our colleagues have followed up with continuing and close collaboration and additional studies to address unanswered questions as well as new questions raised by the already-published ancient DNA findings.

Most recently, we have been working on our third report of ancient DNA from Vanuatu, focusing on questions raised but not answered in Skoglund et al. (2016), Lipson et al. (2018), and Posth et al. (2018a). One such question is: Can we use genetic data to gain insight into the prehistory of the ‘Polynesian Outlier’ islands in geographical Melanesia, where people speak Polynesian languages today? These islands almost always present evidence of initial human occupation much earlier than any possible date of the introduction of Polynesian subgroup languages to them, presumably representing westward gene flow into them from Polynesian populations. A related question is about the effect of post-1000 BP gene flow from Polynesia on islands whose earlier languages were not replaced, but which show Polynesian influence linguistically through borrowings, and culturally in their material and non-material culture.

Instances of Polynesian cultural influence without wholesale language replacement are particularly pertinent with respect to interpreting the oral traditions relating to the *Chief Roi Mata’s Domain* UNESCO World Heritage Site in Vanuatu, where local villagers have a series of stories of his political importance on the island of Efate and neighboring islands (Garanger 1972; Espirat et al. 1973). To explore these issues, the archaeologists on our team consulted with the local community organization representing stakeholders in Chief Roi Mata’s Domain (Ni-Vanuatu people who have a tradition of descent from the community that Chief Roi Mata led). After the community expressed interest and gave consent to analyze skeletons from the burial complex on the small island of Eretok off Efate which was Roi Mata’s traditional burial place and from the village of Mangaasi which was his traditional village, we approached the Musée de l’Homme in Paris to sample skeletal remains in their care. Involvement of the local community is continuing as we analyse and write up the results.

For our current study, the hypotheses being addressed have been formulated entirely by the archaeologists. The hypotheses were motivated by archaeological questions raised by earlier genetic results, and in the case of Chief Roi Mata’s Domain by interest on the part of the local community and chiefs. Such dialogue between geneticists and archaeologists, and with descendant communities where identifiable, is what is needed to put ancient DNA into the service of archaeology and also into the hands of interested local communities with a keen appreciation for their own history. Our experience with ancient DNA from Vanuatu has been a positive example of such dialogue, producing significant findings that meaningfully constrain our understanding of the past and are leading to ever-deeper engagement between archaeology and genetics and, in our most recent work, Indigenous oral traditions.

The ancient DNA research program in Vanuatu has also been significant for archaeology beyond its specific findings concerning the history of the Pacific Islands. It exemplifies a major theme that has emerged from the whole genome ancient DNA revolution in the last few years, which is that large-scale movements of people—involving population turnover and major admixture—have played an important role in a number of important shifts in material culture that are evident in the archaeological record. Since the Second World War, archaeologists have often become wary of claims for prehistoric movements of people because of an association with the ideas of Gustav Kossinna, later integrated into Nazi race theory (Heyd 2017). Instead, cultural diffusion and/or independent invention driven by parallel adaptations to similar environmental changes have been highlighted by many archaeologists as the most plausible primary causes leading to the introduction of new material culture and new cultural practices. The ancient DNA results of the last five years have moved the dial on this debate by showing that large-scale movements of people were in fact far more common in prehistory than many had recently supposed and did accompany major cultural shifts, as was clearly the case in Vanuatu.

Equally important, the whole genome ancient DNA revolution has highlighted the complexities of the past by revealing equally striking cases where important aspects of culture did not always change with movement of people. This occurred for example in Vanuatu, where Austronesian languages ultimately originating in Taiwan became spoken by people of largely Papuan ancestry (Lipson et al. 2018; Posth et al 2018a). It also occurred in Western Europe (Olalde et al. 2018) and South Asia (Narasimhan et al. 2019) where large-scale gene flows from people with ancestry ultimately deriving from the Eurasian Steppe occurred without evidence of large-scale importation into the region of Steppe material culture.

It is interesting to consider how archaeological explanations will change in the aftermath of the whole genome ancient DNA revolution. One prediction is that it will become widely accepted that the documentation of movement of people, although important to understand, does not by itself provide a sufficient explanation for what occurred in prehistory. To know whether or not people moved is of course very necessary in order to make sense of prehistoric events, but equally important is the mechanism for how movement of people contributed to change. The point was made in another way by Johanssen et al. (2017:1119): ‘aDNA evidence of admixture, and perhaps even migration, is important not because it provides an explanation of cultural change. Rather, it is important because it provokes additional, more significant questions, such as what processes may have triggered movements of people, how these movements unfolded, and what the broader social and economic consequences were for the populations involved’.

The true value of genetic studies in documenting movements of people in prehistory is in fact more subtle than it might at first seem. When a large-scale movements of people connecting two regions are documented (such as from the Steppe to Europe at the end of the Neolithic, or the Bismarck Archipelago to Remote Oceania in the late Lapita period), then we can be certain that they occurred on a sufficient scale that cultural influences could have accompanied them. When genetics shows that two areas have been connected by movement of people, this reinforces the plausibility of archaeological models that highlight connections between these areas. Genetic findings can highlight specific connections between ancient locations that may not previously have received as much consideration, indicating places and highlighting hypotheses that can be enriched by further archaeological investigation and evidence. For instance, Lipson et al. (2018) and Posth et al. (2018a) both pointed directly to the island of New Britain in the Bismarck Archipelago as the source for the Papuan genetic ancestry found in Vanuatu.

A Bismarck Archipelago to Vanuatu connection had already been argued for Lapita, leapfrogging the main Solomon Islands chain, based entirely on (at the time contentious) archaeological and linguistic evidence (Sheppard 2011; Walter and Sheppard 2017).^6^ Genetically this movement of people was associated with the First Remote Oceanians, who in Vanuatu are directly tied to the Lapita culture via DNA extracted from human remains found with Lapita pots. The fact that the slightly later movement of Papuans originated in the same archipelago and also leapfrogged the Solomons would be all the more remarkable if it was not directly connected to the first movement of people. We know of no evidence either archaeologically or genetically of immediately post-Lapita connections between Vanuatu and the nearest archipelago to the north – the main Solomons chain. This would suggest that the eastward spread of Papuan ancestry represented a continuing migration stream but in changed circumstances. Discussing similar migration streams, Anthony (1990:904) has commented: ‘Kinship linkages, dependence, and the reduction of obstacles may attract a secondary flow that is quite different in goal orientation and composition from the initial migrant group’. Such phenomena, as with ‘leapfrogging’, are commonly associated with both past and current migrations (Anthony 1990).

Posth et al. (2018a) noted some aspects of culture almost uniquely shared between present-day New Britain and some ethnographically recorded Vanuatu cultures: ‘large nasal-piercing ornaments, penis sheaths, head binding and the rearing of full-circle tusker pigs’ (2018a:736). The raising of pigs with full-circle tusks by knocking out the upper incisors to let the lower ones grow round in a circle is particularly distinctive, as it is shared between only these two areas in the Pacific. In the absence of genetic evidence, archaeologists had no context in which to fit the apparent connection. Similarly, with aspects of Lapita iconography we sometimes find detailed parallels between Lapita designs and those found ethnographically in Taiwan and archaeologically in southern China (Spriggs 2019). These would now appear to be culturally significant results of durable practices rather than long-distance coincidences, given the Taiwanese genetic ties of early Lapita individuals in Vanuatu.

## The Vanuatu Ancient DNA Studies in Broader Context

The positive collaborations between archaeologists and geneticists that have characterized the ancient DNA research in Vanuatu is an example of a type of close collaborative study between archaeologists and geneticists that is unfolding in many world regions, and meaningfully drawing together the two communities one study at a time. We highlight two additional examples in which one of us (D.R.) has actively participated:

### 1. Impacts of Steppe pastoralist expansions on Western and Southern Eurasia

In the 1990s a ‘new synthesis’ for the Neolithic peopling of Western Eurasia became popular, combining archaeology and linguistics (Renfrew 1992), but the data brought to bear in favour of it unravelled spectacularly in light of new genetic evidence (Vander Linden 2016:715). Ancient DNA studies in the last five years, unfolding over multiple papers in multiple laboratories all of which represent committed collaborations between geneticists and archaeologists, have shown that it is unlikely that the Indo-European languages spoken in Europe and South Asia today descend from languages that spread there along with the spread of farming. Instead, large-scale gene flow from the Steppe north of the Black and Caspian Seas occurred after the spread of farming, connecting ancient peoples whose geographic distributions correlate strongly to the reconstructed phylogenetic relationships among Indo-European languages. This provides evidence that it is these ancient movements of people that spread the Indo-European languages spoken today (Haak et al. 2015; Allentoft et al. 2015; Damgaard et al. 2018; Narasimhan et al. 2019; Shinde et al. 2019). In the laboratory of one us (D.R.), we are following up these findings through further close collaborations with archaeologists, exploring how Steppe pastoralist-related ancestry did or did not spread—and how it did or did not correlate to Indo-European languages—in regional studies in Iberia (Olalde et al. 2019), the western Mediterranean islands, southeast Mediterranean, Armenia, the Hungarian plain, and Iron Age Britain (the unreferenced studies all represent work in preparation). Multiple other archaeologist-geneticist teams are also productively exploring related questions.

### 2. Genetic correlates of the spread of the first food producers of East Africa

One of us, D.R., has also been involved in a collaboration with East African archaeologists. The first paper that emerged from that collaboration published data from 16 pre-historic sub-Saharan Africans (Skoglund et al. 2017). It included data from a ~3000BP infant buried with artefacts of the Pastoral Neolithic material culture, whose ancient DNA showed that they were part of a population that made a major demographic impact on peoples of the continent from Ethiopia to southern African along with the spread of pastoralism. Following this study, D.R. initiated a collaboration with one of the study’s co-authors, Mary Prendergast, which has resulted in reporting of genome-wide data from 41 ancient individuals from Kenya and Tanzania most of whom were also from this early pastoralist community and provided a sample size sufficient to reveal the dynamics of formation of this population. The findings included the discovery of an initial mixture between two ancient northeast African populations ~6000–5000BP, followed by their mixture with local East African hunter-gatherers by ~4000–3000BP to produce a stable genetic profile that persisted with negligible mixing in East Africa for about two thousand years (Prendergast et al. 2019). This collaboration has been one in which the archaeologists have been full and equal partners, just like our work in Vanuatu. For example, an archaeologist was first author of the primary paper reporting the findings, and the work also stimulated independent papers by the archaeologists involved (Prendergast and Sawchuk 2018; Sawchuk and Prendergast 2019). As was the case for D.R.’s work concerning Steppe pastoralist migrations, and our projects in Vanuatu, this collaborative team is continuing with further research.

## Conclusion

Rather than disempowering archaeologists, the contribution of ancient DNA has in fact been a liberating experience. Controversies that could never have been resolved solely using archaeological methods (or by DNA studies of present-day populations) have now been settled. It is reasonable to expect that the demographic history of human movements across the Pacific, a topic of debate for centuries, will in the near future be richly understood.

But as we have pointed to here, reconstructing the history of human population movements does not by itself provide a full explanation of anything. Instead, solving demographic issues about the deep past using ancient DNA has allowed the posing of more sophisticated questions than could be asked before, and makes possible multi-disciplinary syntheses that would not otherwise be possible. These syntheses can meaningfully address the nature of long-distance connections between groups, the sociopolitical context in which movements of people occurred, and the effect on relations among groups of changing demographic, linguistic and cultural landscapes. Without the extraordinary power that genome-wide ancient DNA studies provide to understand who moved, when, and how—in the Pacific and elsewhere—it would be far more difficult to understand our past.

## Figures and Tables

**Figure 1:**
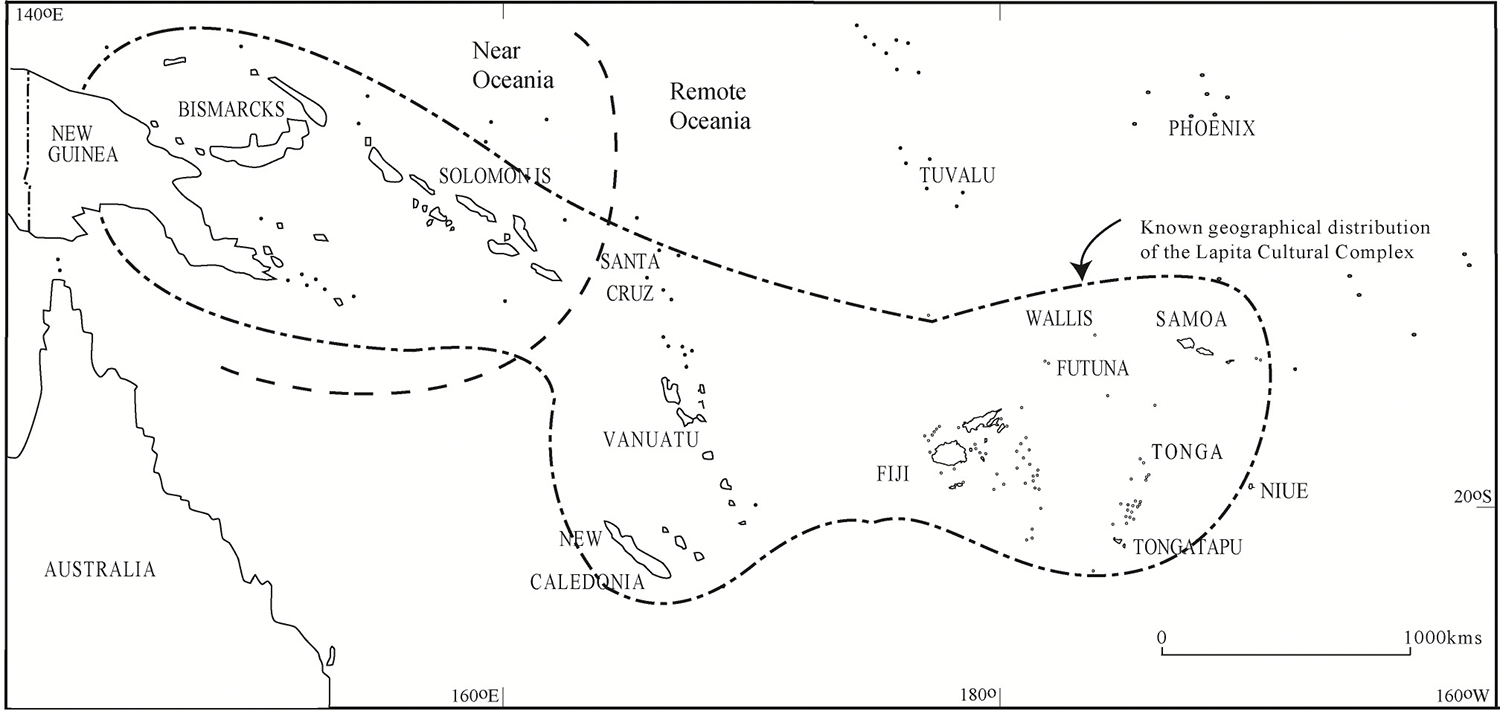
The distribution of the Lapita culture in the Western Pacific. The boundary between Near and Remote Oceania is indicated. Map courtesy of Stuart Bedford.

**Figure 2:**
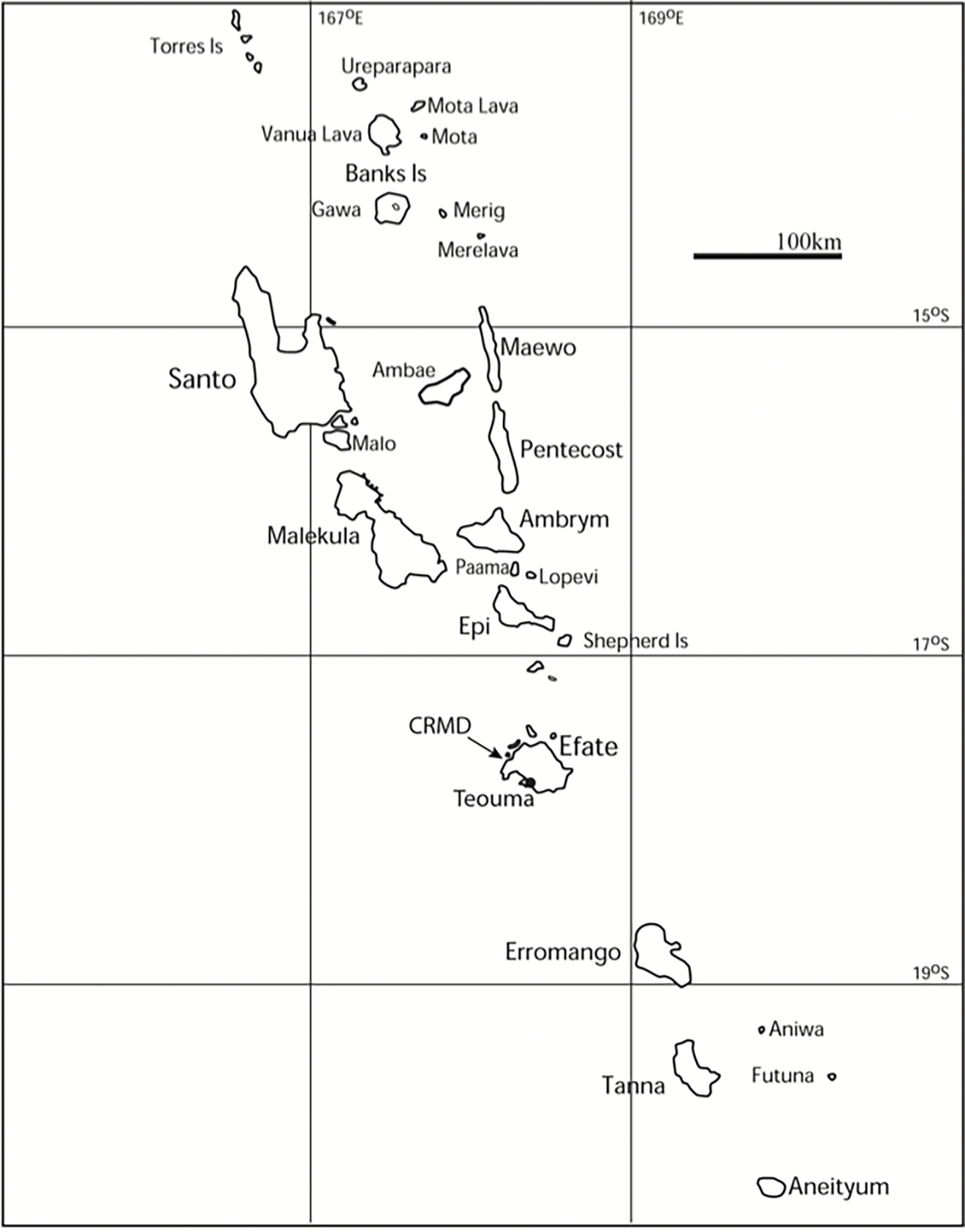
Map of Vanuatu showing the main islands mentioned in the text. Map courtesy of Stuart Bedford.

## Endnotes

1 More than 55 publications relating to Teouma have now been published since 2004. These include specialist analyses as well as at least 12 postgraduate theses, in collaborations including more than 100 scholars from 15 countries and 44 institutions (and many more when including the Skoglund et al. 2016 and Lipson et al 2018 authors).

2 The laboratories that attempted these analyses were at Binghamton University (USA), Auckland University (New Zealand) and Adelaide University (Australia). M.S. thanks the teams led by Koji Lum, Lisa Matisoo-Smith and Alan Cooper for their efforts.

3 This refers to ongoing Australian Research Council-funded work of Hannah James, Wendy Lees and Andrea Ulrichsen in projects led by Rainer Grün, now of Griffiths University, and Catherine Frieman and M.S. at The Australian National University.

4 Examples where other ancient DNA laboratories have contributed data to studies led by one of us (D.R.) include Skoglund et al. 2016, Fu et al. (2016), Skoglund et al. (2017), Mathieson et al. (2018), Olalde et al. (2018)and Posth et al. (2018b). Examples where D.R.’s lab has contributed data to studies led by another group include Lazaridis et al. (2017), Wang et al. (2018), Mittnik et al. (2018), Flegontov et al. (2019), and Brace et al. (2019).

5 We acknowledge that it is hard to come up with a fully satisfactory naming system for genetic groups, and indeed point out that there are also potential problems even with our own preferred term ‘First Remote Oceanian ancestry’ in that it assumes without proof that people of almost entirely East Asian-related ancestry were the first people to live in Remote Oceania (in a similar way, the term ’First American ancestry’ used in studies like Reich et al. (2012) assumes without proof that people of an ancestry profile continuous with most present-day Native Americans were the first humans to reach the Americas). It continues to be possible in theory (although in light of current data we believe relatively unlikely) that at least a few people with Papuan ancestry lived in Remote Oceania in Early Lapita times and simply have not yet been sampled.

6 For instance, in a paper written just before the Skoglund et al. (2016) results became available, Bedford, Spriggs and Shing (2016) expressed strong scepticism of Walter and Sheppard’s arguments for ‘leapfrogging’, based on geomorphological factors and a history of limited research across much of the main Solomons chain. The arguments made there remain cogent, but their scepticism is now much abated.
